# Effect of immune infiltration intensity on the efficacy of neoadjuvant immunotherapy for esophageal cancer

**DOI:** 10.3389/fimmu.2025.1543283

**Published:** 2025-06-12

**Authors:** Yong Zhang, Xinyao Xu, Xiaorong Mu, Juzheng Wang, Jipeng Zhang, Guangyu Xiang, Jiahe Li, Chunlong Zheng, Huaiyu Wang, Qiang Lu

**Affiliations:** ^1^ Department of Thoracic Surgery, Tangdu Hospital, Air Force Medical University, Xi’an, China; ^2^ College of Life Sciences, Northwest University, Xi’an, China; ^3^ Department of Pathology, Tangdu Hospital, Air Force Medical University, Xi’an, China; ^4^ Department of Pharmacy, Tangdu Hospital, Air Force Medical University, Xi’an, China; ^5^ Department of Thoracic Surgery, The First People’s Hospital of Xianyang, Xianyang, China; ^6^ Basic Medical College, Air Force Medical University, Xi’an, China; ^7^ Department of Thoracic Surgery, Air Force Medical Center, Xi’an, China

**Keywords:** immune infiltration, neoadjuvant immunotherapy, esophageal cancer, PD-1, CXCL10

## Abstract

**Background:**

Esophageal squamous cell carcinoma (ESCC) treatment often involves neoadjuvant therapy combining chemotherapy and immune checkpoint inhibitors. However, the effectiveness of these treatments is limited by immune infiltration in the tumor microenvironment.

**Methods:**

We analyzed single-cell transcriptomic data from 22 patients with resectable ESCC, collected before and after neoadjuvant therapy. Differences in gene expression between patients achieving a complete pathological response (pCR) and those who did not were assessed. We further validated our findings using RNAseq data from The Cancer Genome Atlas (TCGA), and conducted quantitative qRT-PCR and Western blot analyses on tumor tissues from a clinical cohort.

**Results:**

Significant differences in gene expression related to T cell activation, natural killer cell activity, and cytokine signaling were observed between pCR and non-pCR patients. Notable genes included CXCL10, CXCL11, ME1, MT1X, FAT1, OAS2, and MT2A. TCGA data confirmed a correlation between high gene expression and increased tumor mutational burden as well as improved survival rates, particularly for CXCL10. qRT-PCR revealed significant upregulation of CXCL10, CXCL11, ME1, MT1X, FAT1, OAS2, and MT2A in tumor tissues compared to normal tissues. Western blot analysis showed increased protein levels of CXCL10, CXCL11, OAS2, MT1E, and MT1X, while FAT1 was downregulated.

**Conclusion:**

Our study highlights the critical role of immune infiltration and associated molecular pathways in the efficacy of neoadjuvant immunotherapy for ESCC. Specific genes, such as CXCL10, are promising as predictive markers for treatment response and survival.

## 1 Introduction

Esophageal squamous cell carcinoma (ESCC) represents the predominant histologic type globally, particularly prevalent in developing countries. Despite incremental advances in diagnostics and therapeutics, ESCC presents a grim 5-year survival rate ranging from 12-20% (1). The standard treatment for locally advanced ESCC involves neoadjuvant chemoradiotherapy combined with surgery, following the success of the CROSS trial (2). However, this approach can heighten toxicity levels, leading to severe side effects such as pneumonia and myocardial injury (3).

In recent years, the integration of immune checkpoint inhibitors (ICIs), targeting programmed cell death 1 (PD-1) and its ligand PD-L1, has emerged as a promising neoadjuvant treatment strategy for early-stage solid tumors, including breast cancer, lung cancer, and ESCC (4, 5). Clinical trials such as KEYNOTE 590 and CheckMate 649 have shown promising antitumor activity and safety of immunotherapies with or without chemotherapy in advanced ESCC patients (6, 7). These results provide a strong rationale for utilizing ICIs in the preoperative treatment setting for ESCC.

Our study aims to investigate the impact of immune infiltration-related genes on the efficacy of neoadjuvant immunotherapy for esophageal cancer. Through the SCALE-1 exploratory study (8), we conducted an in-depth analysis of biomarkers associated with PD-1 treatment response. We found distinct gene expression profiles between patients achieving pathological complete response (pCR) and those without pCR, indicating the potential role of immune-related genes in treatment outcomes. Notably, genes involved in T cell activation, natural killer cell activity, cytokine and chemokine signaling, and IFN-γ response pathways showed significant differential expression, suggesting their importance in modulating the tumor microenvironment.

Furthermore, integrating our findings with data from The Cancer Genome Atlas (TCGA) provided additional insights into the molecular landscape of esophageal cancer. Consistent upregulation of identified genes in esophageal cancer samples, along with their association with higher tumor mutational burden (9) and survival rates, supports their consideration as potential predictive markers for immunotherapy response.

In summary, our study highlights the critical role of immune infiltration intensity and its molecular determinant — *CXCL10* in shaping the efficacy of neoadjuvant immunotherapy for esophageal cancer. These findings deepen our understanding of tumor-immune interactions and offer implications for personalized treatment strategies in this challenging malignancy.

## 2 Materials and methods

### 2.1 The origin of single-cell RNA sequencing data

The data for our article were derived from the single-cell data of the previous study (10) and subjected to conventional cell annotation analysis based on the methods outlined in the previous article.

### 2.2 scRNA-seq data processing

Based on scRNA-seq data and H&E pathological examination results from esophageal squamous cell carcinoma (ESCC) patients undergoing neoadjuvant chemoimmunotherapy, we categorized pre-treatment (T_B) and post-treatment (T_A) ESCC tumors into three distinct groups: pathological complete response (pCR_T_B, pCR_T_A), major pathological response (MPR_T_B, MPR_T_A), and incomplete pathological response (IPR_T_B, IPR_T_A) groups. Combined with the previous comprehensive analysis of the tumor immune microenvironment (TIME) (using the data in **Appendix Table A1** of the SCALE-1 trial, which lists 289 immune-related genes), we used the R package “Seurat” (11) to perform differential expression analysis and obtained 9 genes (*CXCL10, CXCL11, MAGEA1, OAS2, CD209, CD27, CD79A, KLRB1*, and *TNFRSF17*) (12–18).

### 2.3 Analyzing the expression of key 9 genes based on ESCA sample data from TCGA

We obtained 182 esophageal carcinoma (ESCA) samples and 13 normal samples from the TCGA database (https://portal.gdc.cancer.gov/). Comparing normal samples to ESCA samples, we analyzed the mRNA expression levels of *CXCL10*, *CXCL11*, *MAGEA1*, *OAS2*, *CD209*, *CD79A*, *KLRB1*, and *TNFRSF17* in ESCA cases using the R package “DESeq2” (19).

### 2.4 Analysis of tumor mutation burden about those key 9 genes

Mutation data of ESCA cases were retrieved from the TCGA database, and tumor mutational burden (TMB) was calculated using the R package “maftools” (20). Pearson correlation analysis (21) was employed to examine the relationship among *CXCL10*, *CXCL11*, *MAGEA1*, *OAS2*, *CD209*, *CD27*, *CD79A*, *KLRB1*, and *TNFRSF17*.

### 2.5 Survival curve analysis

In this study, 182 ESCA cases were stratified into high-expression and low-expression groups based on the median values of *CXCL10*, *CXCL11*, *MAGEA1*, *OAS2*, *CD209*, *CD27*, *CD79A*, *KLRB1*, and *TNFRSF17* mRNA expression. Kaplan-Meier survival curves (22) were generated using the R package “survminer”.

### 2.6 Analysis of half maximal inhibitory concentration (IC50)

The Cyclopamine sensitivity (23) of CXCL10 in the high-expression and low-expression groups was analyzed using the GEPIA2 (24) web tool (http://gepia2.cancer-pku.cn/#index), and relevant sensitivity bar graphs were generated.

### 2.7 Real-time quantitative PCR and western blot of nine genes

Total RNA was extracted from culture cells using TRI pure based on the manufacturer’s instructions. The OD value of total RNA was detected and used for subsequent RT-PCR quantification. Then, a Prime Script TM reagent Kit with gDNA Eraser was used to reverse-transcribe 1 µg of total RNA in a 20 µL volume into cDNA. Quantitative real-time PCR was performed using the Quantitative Real-time PCR Kit. All primers were designed and synthesized by Shanghai Integrated Biotech Solutions Co., Ltd. (Shanghai, China) **Supplementary Table S1**. The results were normalized using GAPDH as an internal control. Western blot analysis was conducted as previously described. 6 Samples were probed with anti-genes (9) (Abcam, Cambridge, UK), or anti-actin (Sigma-Aldrich). Densitometric analysis was carried out using Image J software (Version 1.44o; NIH).

### 2.8 Statistical analysis

Statistical analyses were performed using R software (version 4.1.2, https://www.r-project.org/). To compare continuous variables between two groups, an independent Student’s t-test was used for normally distributed data, while the Mann–Whitney U-test was applied for non-normally distributed data. All p-values were calculated using a two-tailed approach, with significance defined as P < 0.05.

## 3 Results

### 3.1 The expression of nine key genes in ESCA before/after neoadjuvant chemoimmunotherapy

Using previously collected single-cell RNA sequencing (scRNA-seq) data from ESCA diseased tissues, we investigated the expression levels of *CXCL10*, *CXCL11*, *MAGEA1*, *OAS2*, *CD209*, *CD79A*, *KLRB1*, and *TNFRSF17* in various groups before and after treatment. Our study revealed that expression of *OAS2* (**Supplementary Figure 1c**) was significantly increased in the IPR_T_B group.

### 3.2 Expression of nine genes in various subtypes of different samples

**Figure 1:**
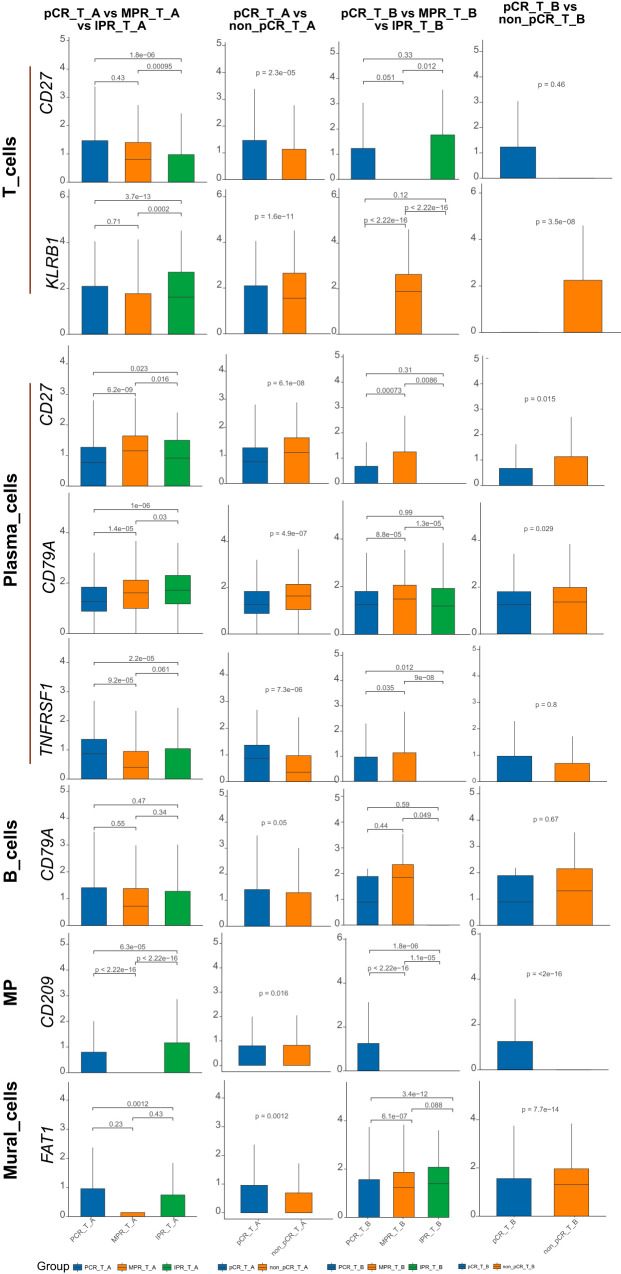
Nine genes expression in ESCA subtype cells before/after neoadjuvant chemoimmunotherapy. CD27, CD79A, FAT1, KLRB1, CD209, CXCL10, OAS2, TNFRSF1 showed significant differential expression across the subtypes in the above barplot. Specifically, in all differential analyses, CD79A exhibited changes in the B_cells subtype. CD209 displayed alterations in both the MP and Mural_cells subtypes. CD27, CD79A, and TNFRSF1 showed changes in the Plasma_cells subtype, while CD27 and KLRB1 exhibited alterations in the T_cells subtype.

**Figure 2:**
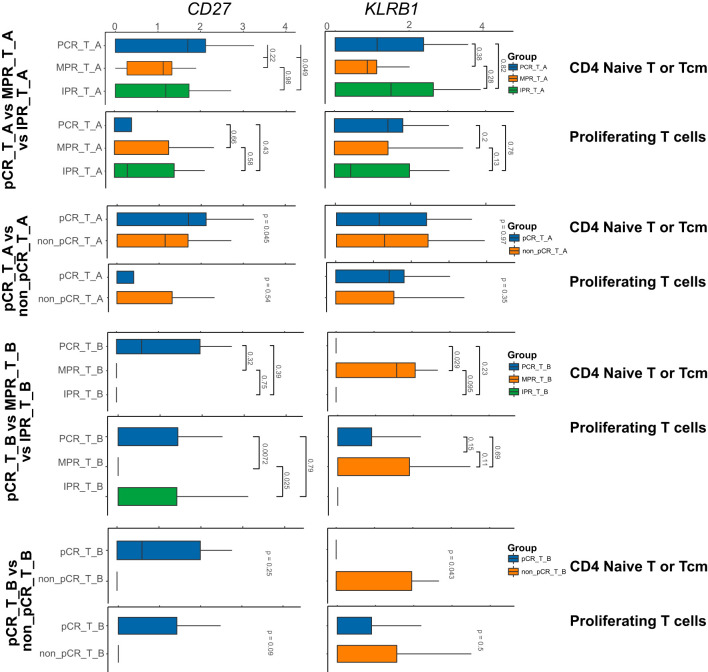
Nine genes expression in the subtype cells of T subtype-cell before/after neoadjuvant chemoimmunotherapy. In the above barplot, CD4_Naive_T and ProlifT subtypes exhibited significant differences in CD27 and KLRB1 expression. In the CD8_exhausted_T subtype, significant differences were observed in CD27, KLRB1, and OAS2 expression. Additionally, except for Treg, KLRB1 was identified as a significantly differentially expressed gene in the remaining subtypes.

Based on early cell subtype annotation, we conducted differential analysis on the same single-cell subtypes across different samples, primarily observing changes in the expression of the nine genes mentioned above. We identified significant differential expression in seven genes: *CD27*, *CD79A*, *KLRB1*, *CD209*, *CXCL10*, *OAS2*, and *TNFRSF1*. Notably, *CD79A* showed consistent changes across all differential analyses in the B_cells subtype (**Figure 1**). Similarly, *CD209* displayed changes in both the MP and Mural_cells subtypes. In the Plasma_cells subtype, *CD27*, *CD79A*, and *TNFRSF1* demonstrated consistent changes. Lastly, in the T_cells subtype, *CD27* and *KLRB1* showed consistent alterations (**Figure 2**).

The expression of *CXCL10* showed significant differences in the MP subtype of PCR_T_B, MPR_T_B, and IPR_T_B samples, with a notable increase in IPR_T_B and extremely low expression in MPR_T_B. This pattern was similarly observed in the MP subtype of PCR_T_B and non_PCR_T_B samples. However, *CXCL10* exhibited a significant increase in expression in the pCR_T_B subtype and extremely low expression in the non_pCR_T_B subtype (**Supplementary Figures 2a, b**).

**Figure 3:**
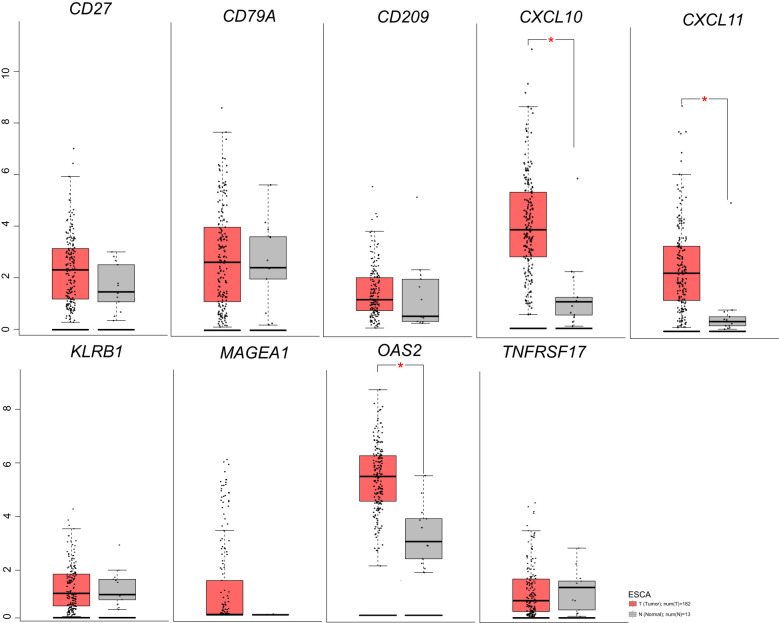
Nine genes expression in ESCA before/after neoadjuvant chemoimmunotherapy based on TGCA database.

We further investigated the expression differences of these genes in the T cell subtypes of different samples through group differential analysis. The results revealed that *CD27*, *KLRB1*, *OAS2*, and *FAT1* exhibited relatively significant changes across various T cell subtypes, with distinct genes showing significant changes in different subtypes. Specifically, *CD27* and *KLRB1* showed significant differences in CD4_Naive_T and ProlifT subtypes, while *CD27*, *KLRB1*, and *OAS2* exhibited significant differences in the CD8_exhausted_T subtype. Additionally, apart from Treg, *KLRB1* was identified as a significant differentially expressed gene in the remaining subtypes (**Figure 3**).

### 3.3 Analysis of nine genes expression in ESCA via the TCGA Database

**Figure 4:**
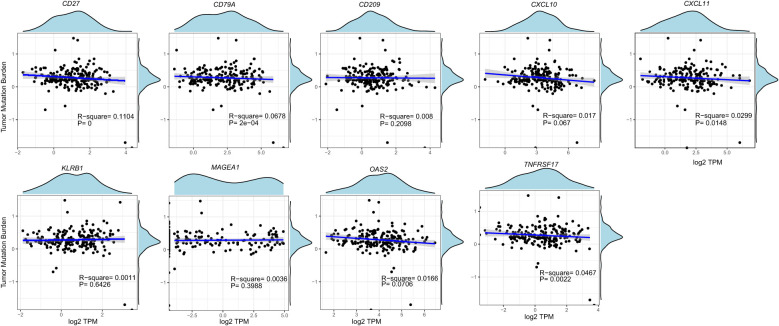
The tumor mutational burden of those nine genes in ESCA based on TGCA database.

We analyzed ESCA data from the TCGA database to investigate the differential expression of nine genes. Our findings revealed an overall increase in the expression of *CXCL10*, *CXCL11*, *MAGEA1*, *OAS2*, *CD209*, *CD27*, *CD79A*, *KLRB1*, and *TNFRSF17* in ESCA patients compared to the normal group (**Figure 3**). Specifically, *CXCL10*, *CXCL11*, and *OAS2* exhibited significant elevation, while the expression of other genes showed a slight increase. Additionally, our analysis unveiled that, except for the *CXCL10* gene, there was no significant correlation between the expression of these genes and the tumor mutational burden (TMB) (**Figure 4**).

### 3.4 The association between the expression of nine genes and prognosis in ESCA

**Figure 5:**
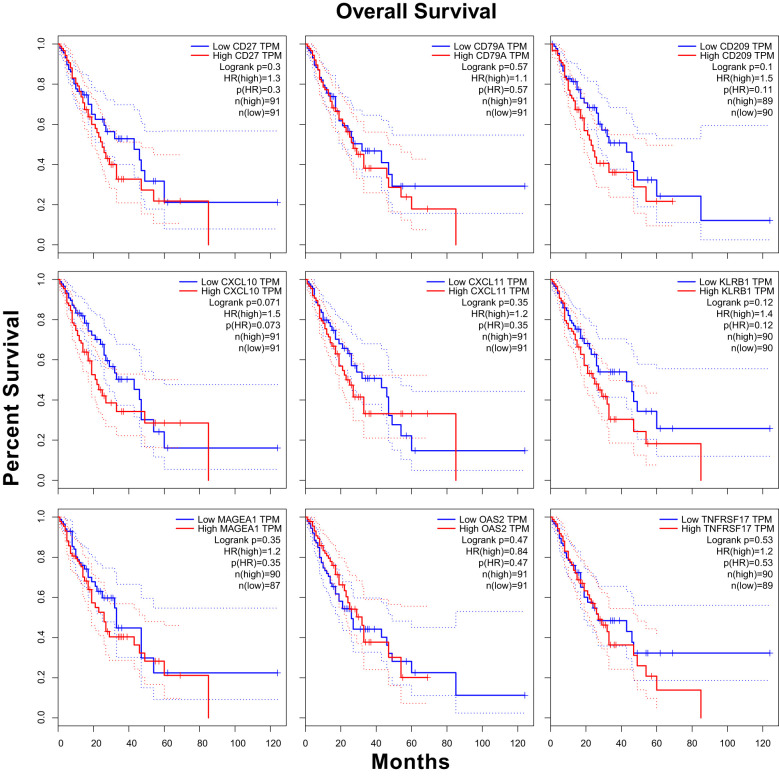
The survival curve of those nine genes in ESCA based on TGCA database.

We conducted survival curve analysis for *CXCL10*, *CXCL11*, *MAGEA1*, *OAS2*, *CD209*, *CD27*, *CD79A*, *KLRB1*, and *TNFRSF17* in a sample of 182 ESCA cases from the TCGA database. Our findings indicated that the group with high *CXCL10* expression exhibited a significantly lower survival probability and relatively poorer prognosis compared to the group with low *CXCL10* expression (HR = 1.5, p(HR) = 0.073) (25, 26) (**Figure 5**).

### 3.5 Analysis of half maximal inhibitory concentration (IC50) on CXCL10 gene

**Figure 6:**
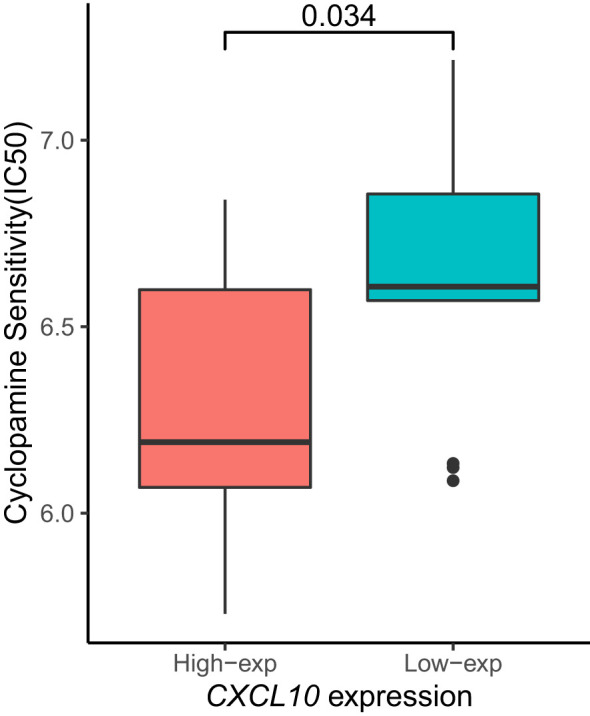
The cyclopamine sensitivity of the CXCL19 gene in ESCA based on TGCA database.

Subsequently, we aimed to investigate the sensitivity of patients stratified by high or low expression of the *CXCL10* gene to specific therapeutic drugs, thus performing an analysis of the half maximal inhibitory concentration (IC50). We found that the disease group with low *CXCL10* expression exhibited higher sensitivity to Cyclopamine (**Figure 6**).

### 3.6 GeneQuantitative PCR and WB to detect the expression of 9 genes

**Figure 7:**
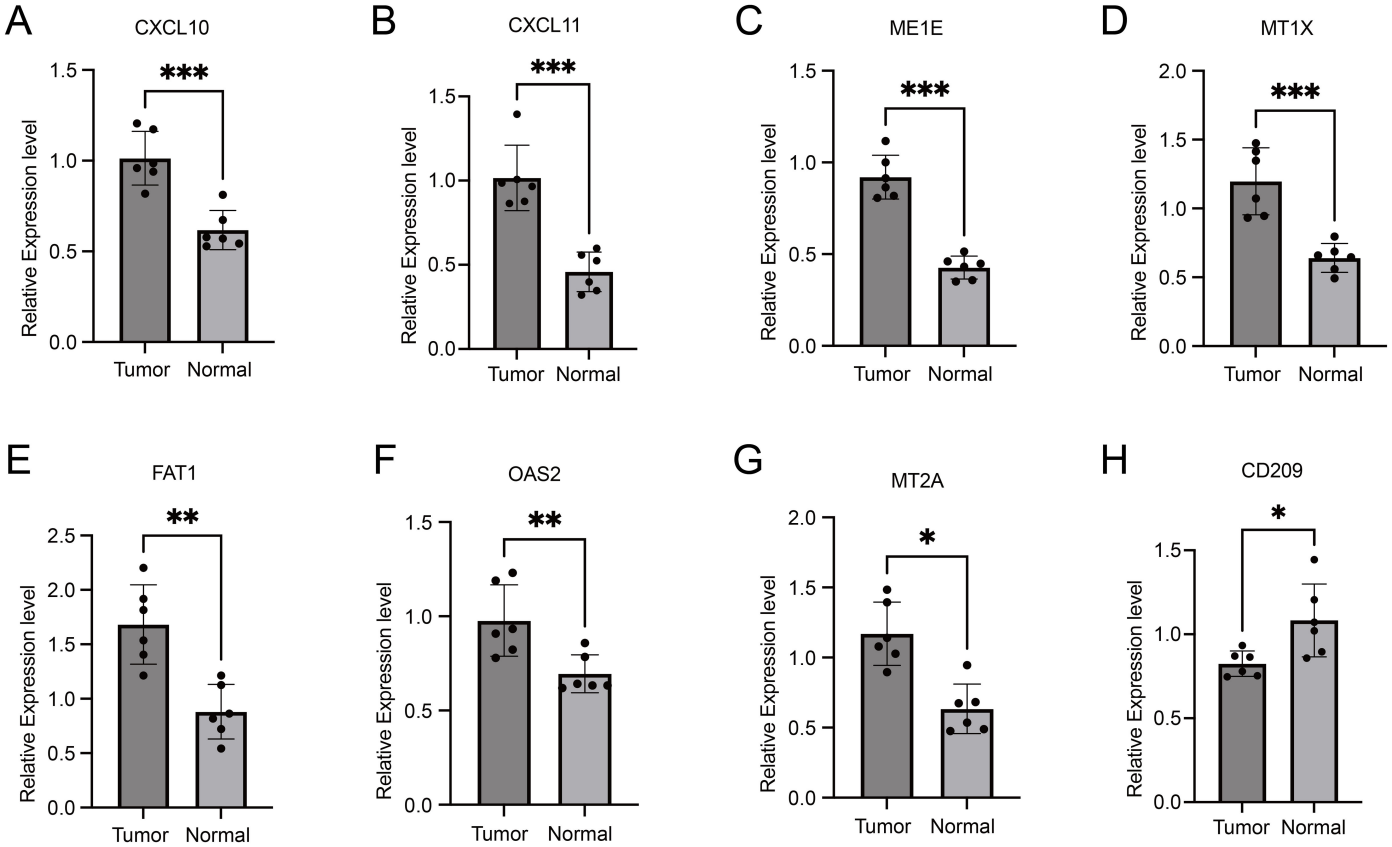
Comparative qRT-PCR analysis of gene expression levels in tumor tissues versus normal tissues. Asterisks indicating levels of significance: ***p < 0.001, **p < 0.01, *p < 0.05.

**Figure 8:**
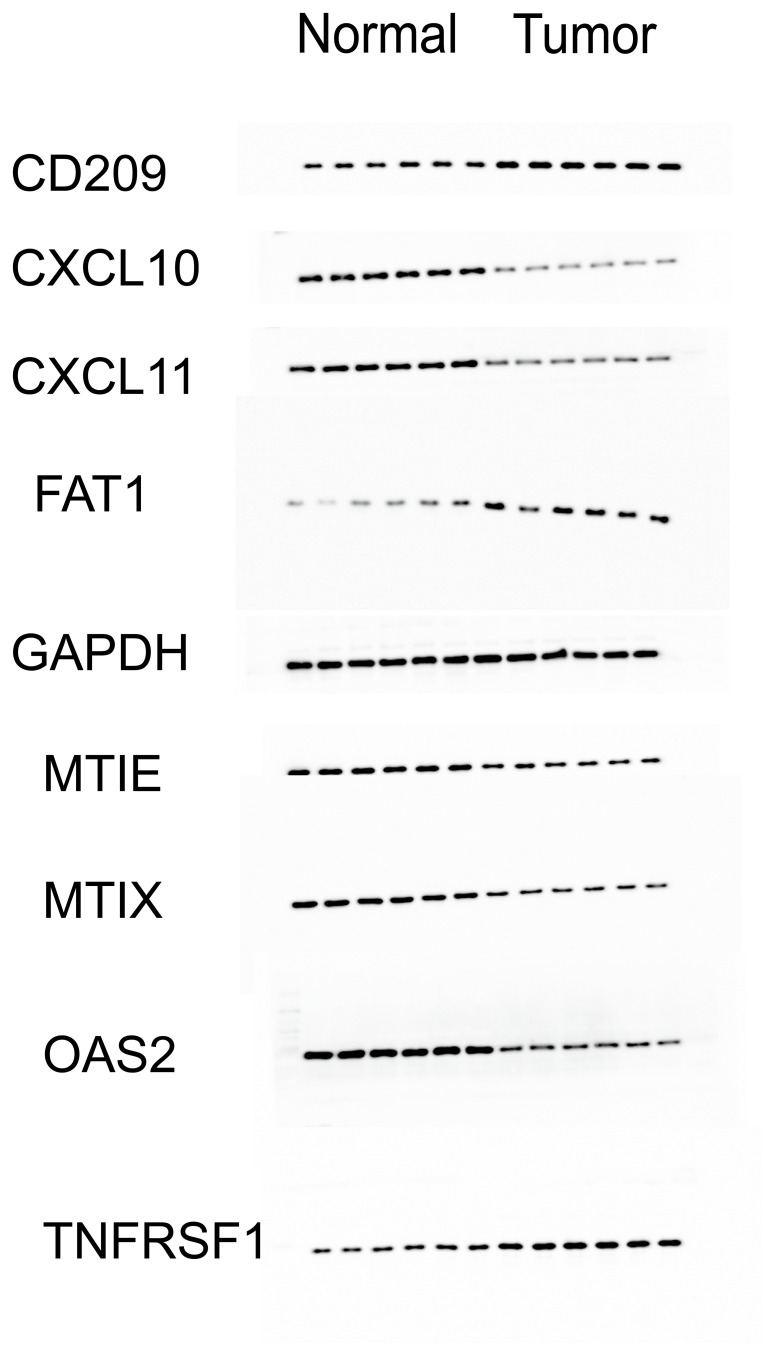
The expression of nine proteins by western blot in normal and tumor tissues. Western blot analysis revealed a significant upregulation of CXCL10, CXCL11, OAS2, MTIE, and MTIX proteins in tumor tissues, which is consistent with the qPCR data. In contrast, FAT1 exhibited a downregulation in tumor tissues, presenting an opposite trend to the qPCR results. The expression of CD209, mirrored the qPCR results, with higher levels observed in normal tissues.

In order to further validate the expression of these nine genes in esophageal cancer, we conducted a study involving clinical cases where we assessed the transcriptome and protein levels of these genes. Our qRT-PCR analysis revealed significant differences in 8 genes between tumor tissues and normal tissues (**Figure 7**), including *CXCL10*, *CXCL11*, *ME1E*, *MT1X*, *FAT1*, *OAS2*, *MT2A* and *CD209*. These findings were consistent across different samples, showing a notable increase in expression levels in tumor tissues compared to normal tissues, except for *CD209* which displayed higher expression in normal tissues. This trend was similarly observed at the protein level, as Western blot results indicated significant upregulation of CXCL10, CXCL11, OAS2, MTIE, and MTIX in tumor tissues, although FAT1 exhibited an opposite trend compared to the qPCR results (**Figure 8**). Notably, CD209 expression mirrored the qPCR results, showing higher levels in normal tissues.

## 4 Discussion

Based on our findings, we propose that *CXCL10* could serve as a potential prognostic marker to guide therapeutic interventions for esophageal cancer. Neoadjuvant therapy, comprising chemotherapy and immune checkpoint inhibitors, represents a promising approach for treating esophageal cancer, particularly squamous cell carcinoma. However, the efficacy of this treatment modality is often compromised by immune infiltration in the tumor microenvironment.

Our study utilized single-cell transcriptomic analysis of resectable esophageal cancer patients before and after neoadjuvant therapy, revealing significant differences in gene expression between those achieving a complete pathological response (pCR) (27) and those who did not. Notably, genes associated with T cell activation, natural killer cell activity, and cytokine signaling exhibited (28–31) substantial alterations, suggesting their potential as predictive markers for treatment response.

Further analysis of RNAseq data from The Cancer Genome Atlas (TCGA) corroborated our findings, indicating a correlation between high gene expression levels, particularly *CXCL10*, with greater tumor mutational burden and improved survival rates. This underscores the importance of considering immune-related gene expression profiles in determining patient prognosis and treatment outcomes.

Moreover, our investigation into the sensitivity of patients with varying *CXCL10* expression levels to specific therapeutic drugs revealed that those with low *CXCL10* expression exhibited increased sensitivity to Cyclopamine. This highlights the potential utility of *CXCL10* expression as a biomarker for predicting treatment response and guiding personalized therapeutic strategies in esophageal cancer.

In conclusion, our study contributes to a deeper understanding of tumor-immune interactions and offers valuable insights for optimizing treatment strategies in esophageal cancer. By identifying CXCL10 as a potential prognostic marker and elucidating its role in therapeutic sensitivity, we pave the way for the development of more tailored and effective treatment approaches for this challenging malignancy.

## Data Availability

The original contributions presented in the study are included in the article/**Supplementary Material**. Further inquiries can be directed to the corresponding author.

## Supplementary material

Supplementary Figure 1
**(a)** Nine genes expression in ESCA samples containing PCR_T_A, MPR_T_A, and IPR_T_A. **(b)** Nine genes expression in ESCA samples containing pCR_T_A, and non_pCR_T_A. **(c)** Nine genes expression in ESCA samples containing PCR_T_B, MPR_T_B, and IPR_T_B. **(d)** Nine genes expression in ESCA samples containing pCR_T_B, and non_pCR_T_B.

Supplementary Figure 2
**(a)** Three genes expression in ESCA subtype cells before/after neoadjuvant chemoimmunotherapy. **(b)** One gene expression in ESCA subtype cells before/after neoadjuvant chemoimmunotherapy.
