# Supplementary material for: Effect of immune infiltration intensity on the efficacy of neoadjuvant immunotherapy for esophageal cancer
Source: Front Immunol. 2025 Jun 12;16:1543283. doi: 10.3389/fimmu.2025.1543283 (PMC12198219; doi:10.3389/fimmu.2025.1543283)
Supplement: Supplementary Figure 1 — (a) Nine genes expression in ESCA samples containing PCR_T_A, MPR_T_A, and IPR_T_A. (b) Nine genes expression in ESCA samples containing pCR_T_A, and non_pCR_T_A. (c) Nine genes expression in ESCA samples containing PCR_T_B, MPR_T_B, and IPR_T_B. (d) Nine genes expression in ESCA samples containing pCR_T_B, and non_pCR_T_B. [file DataSheet1.pdf]

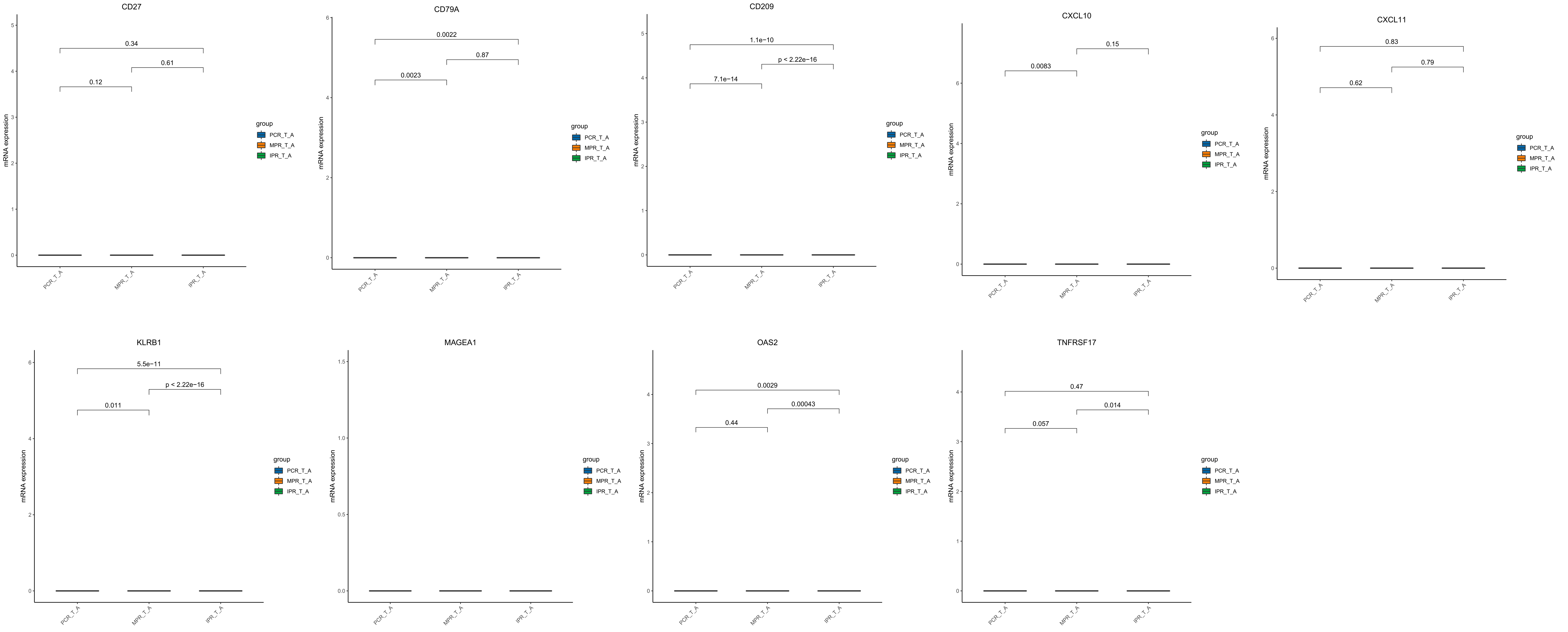

Supplementary FIGURE 1a. Nine genes expression in ESCA samples containing PCR_T_A, MPR_T_A, and IPR_T_A
