# Supplementary figures and images for: Effect of immune infiltration intensity on the efficacy of neoadjuvant immunotherapy for esophageal cancer
Source: Front Immunol. 2025 Jun 12;16:1543283. doi: 10.3389/fimmu.2025.1543283 (PMC12198219; doi:10.3389/fimmu.2025.1543283)

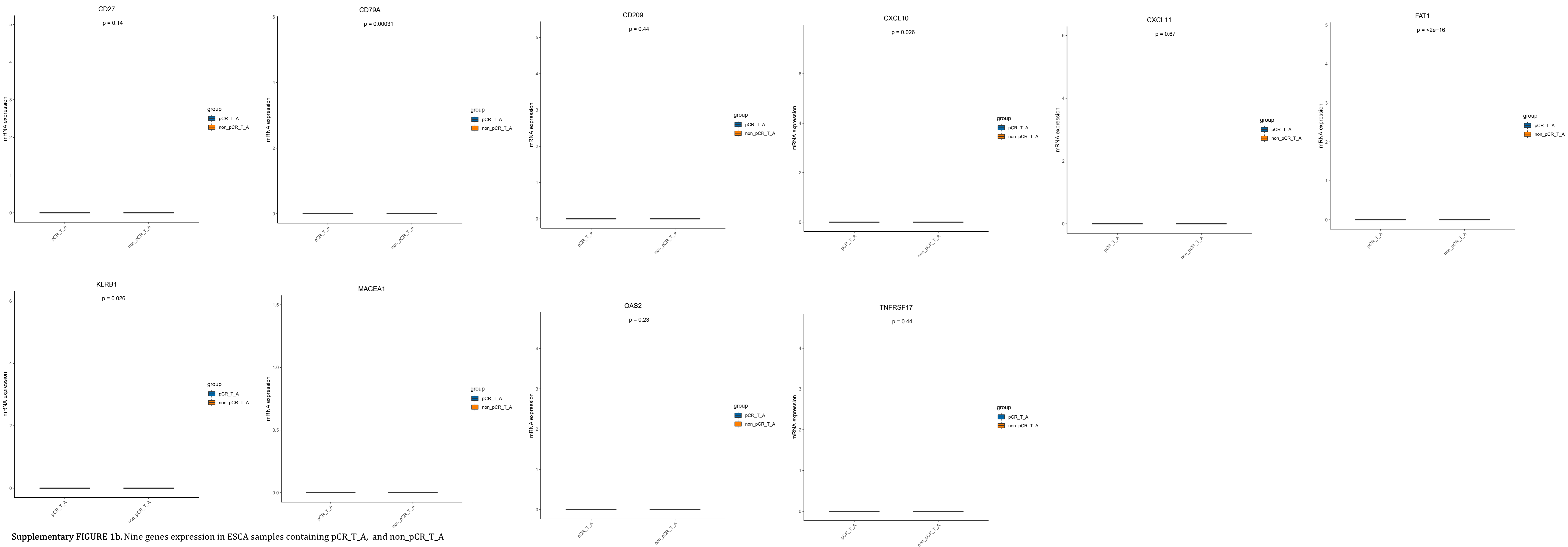

Supplement: Supplementary Figure 2 — (a) Three genes expression in ESCA subtype cells before/after neoadjuvant chemoimmunotherapy. (b) One gene expression in ESCA subtype cells before/after neoadjuvant chemoimmunotherapy. [file DataSheet2.pdf]

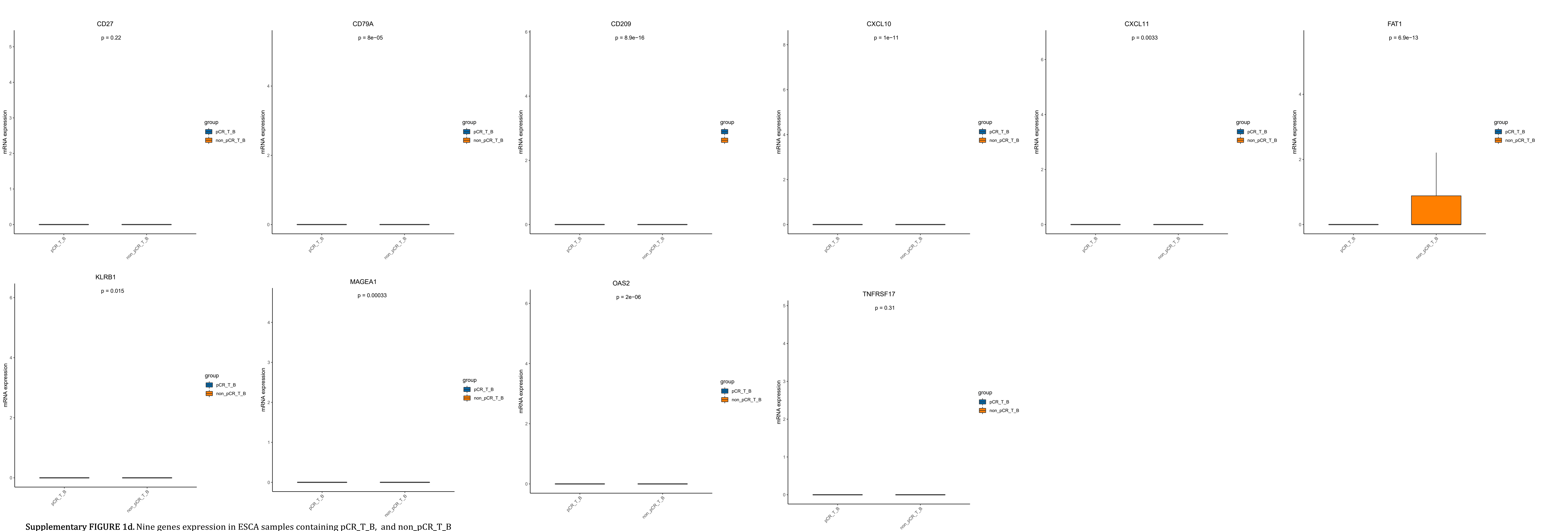

Supplementary FIGURE 1d. Nine genes expression in ESCA samples containing pCR\_T\_B, and non\_pCR\_T\_B

Supplement: Supplementary file 4 [file DataSheet4.pdf]
