# Supplementary material for: Effect of immune infiltration intensity on the efficacy of neoadjuvant immunotherapy for esophageal cancer
Source: Front Immunol. 2025 Jun 12;16:1543283. doi: 10.3389/fimmu.2025.1543283 (PMC12198219; doi:10.3389/fimmu.2025.1543283)
Supplement: Supplementary file 3 [file DataSheet3.pdf]

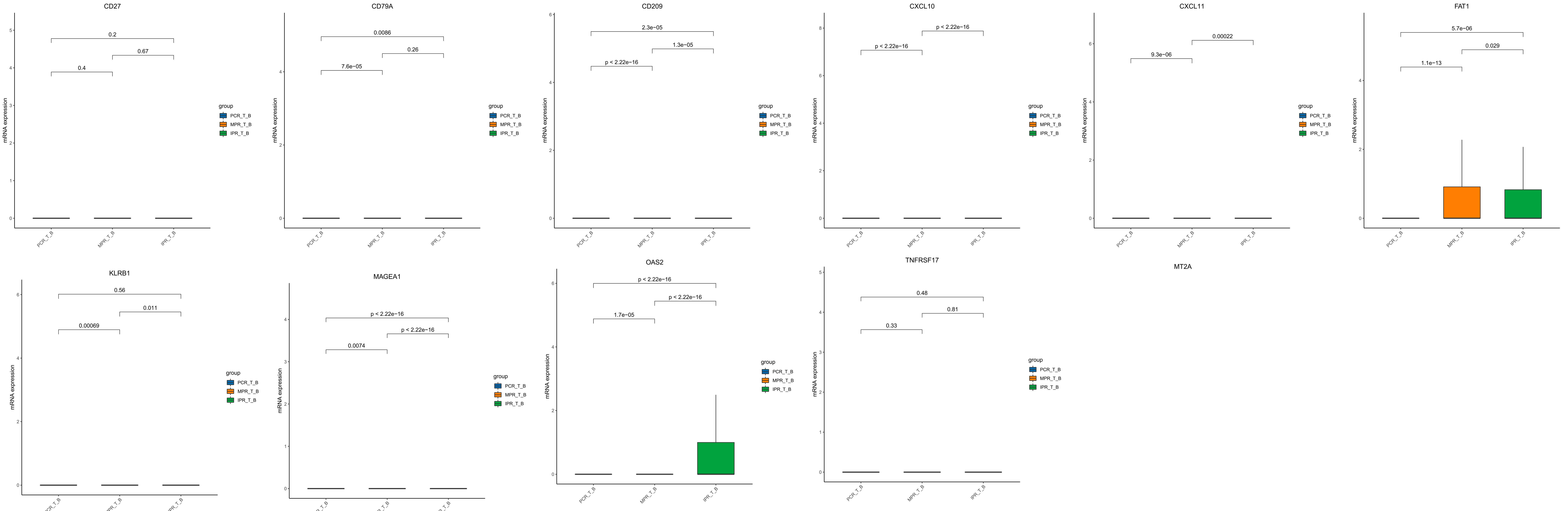


**Supplementary FIGURE 1c.** Nine genes expression in ESCA samples containing PCR_T_B, MPR_T_B, and IPR_T_B
