# Supplementary material for: Effect of immune infiltration intensity on the efficacy of neoadjuvant immunotherapy for esophageal cancer
Source: Front Immunol. 2025 Jun 12;16:1543283. doi: 10.3389/fimmu.2025.1543283 (PMC12198219; doi:10.3389/fimmu.2025.1543283)
Supplement: Supplementary file 6 [file DataSheet6.pdf]

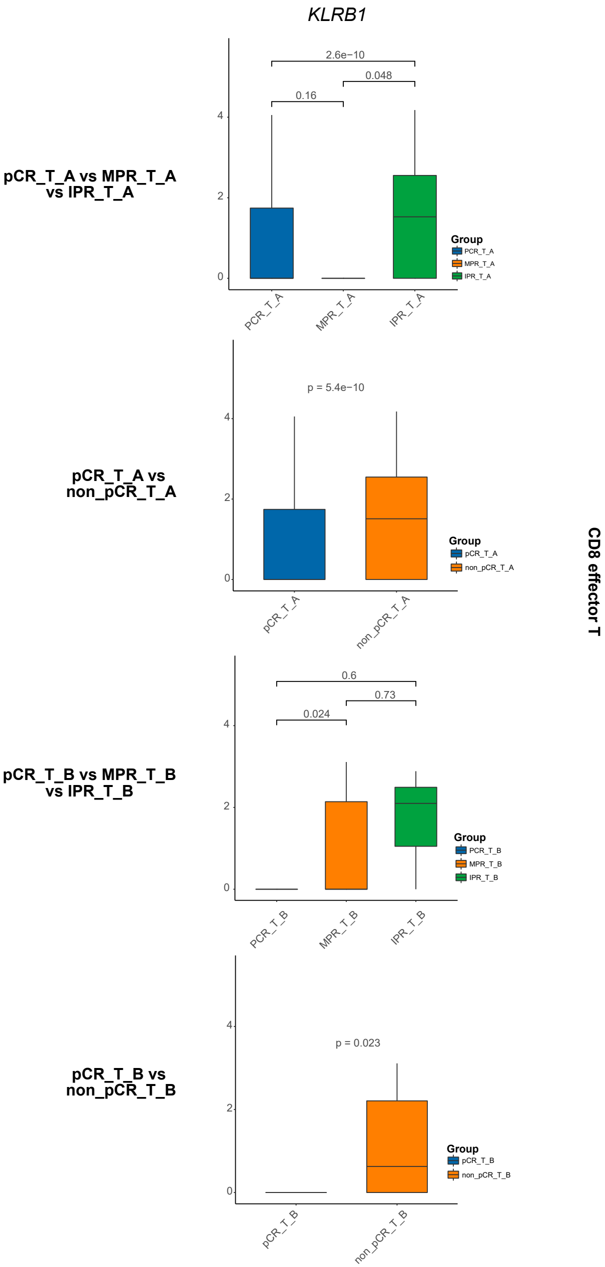

Supplementary FIGURE 2b. One gene expression in ESCA subtype cells before/after neoadjuvant chemoimmunotherapy
