# Supplementary material for: Effect of immune infiltration intensity on the efficacy of neoadjuvant immunotherapy for esophageal cancer
Source: Front Immunol. 2025 Jun 12;16:1543283. doi: 10.3389/fimmu.2025.1543283 (PMC12198219; doi:10.3389/fimmu.2025.1543283)

CD209

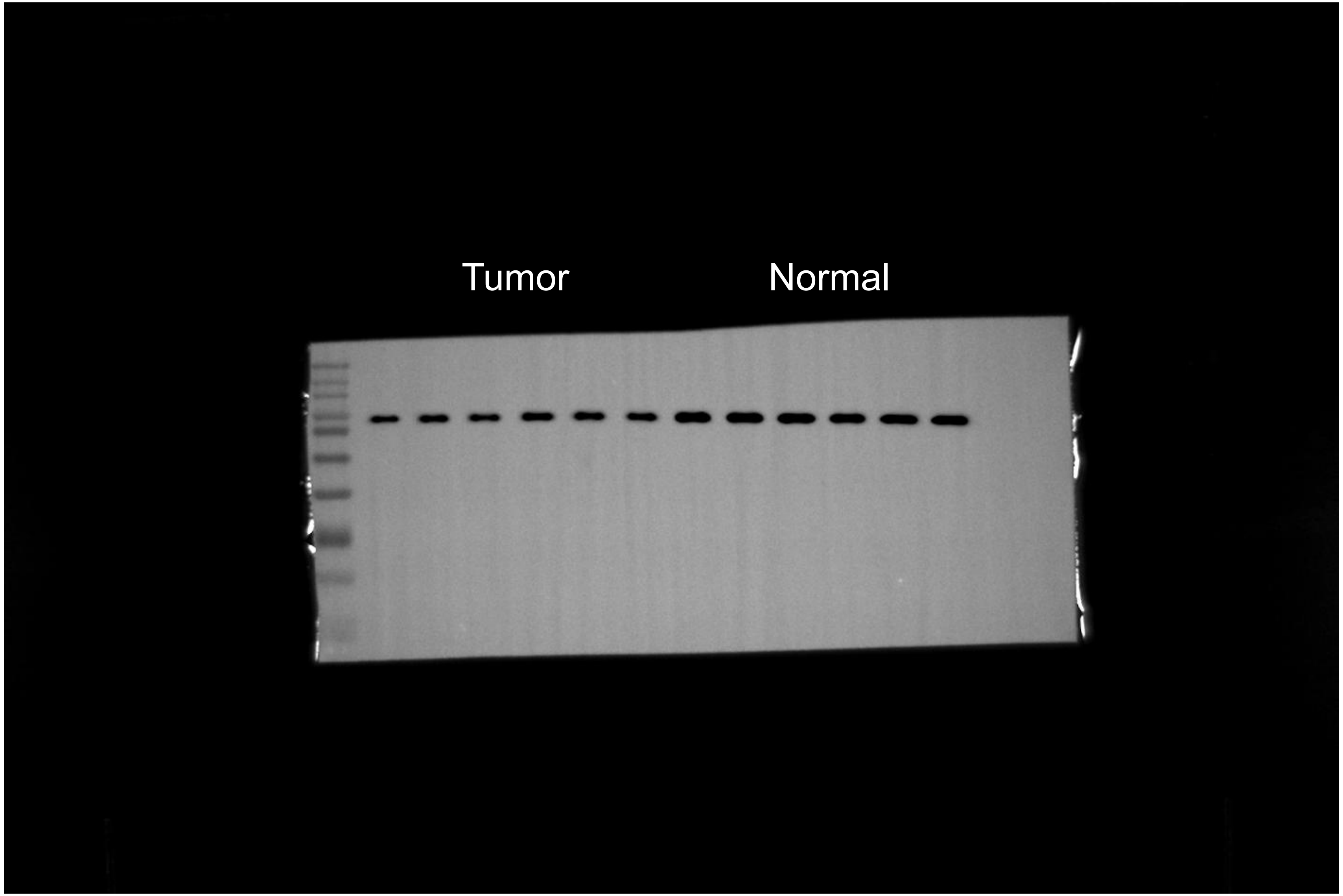

The original Western blot (WB) images are submitted here. The six samples on the left are from tumor tissues, while those on the right are from normal tissues.

CXCL10

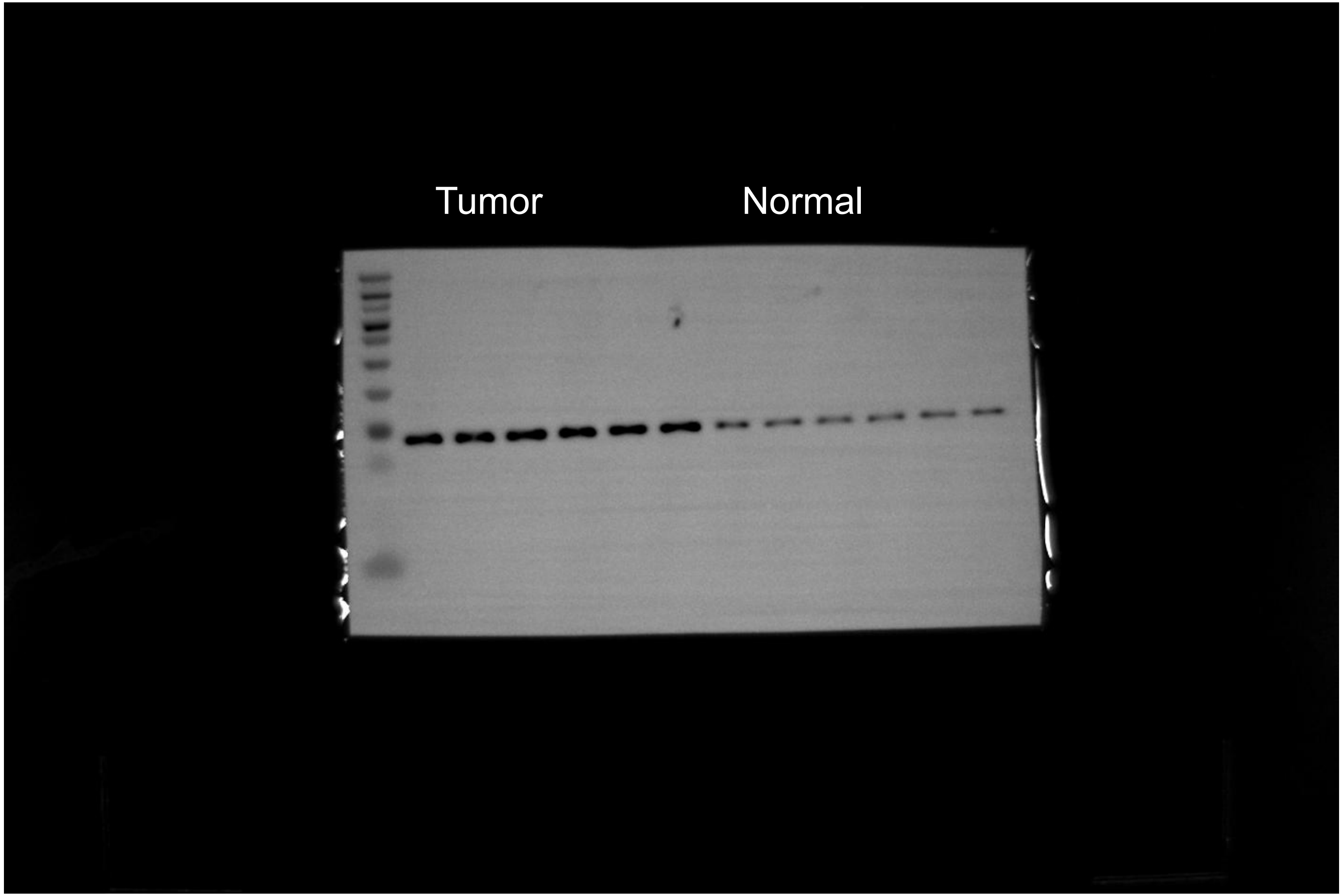

CXCL11

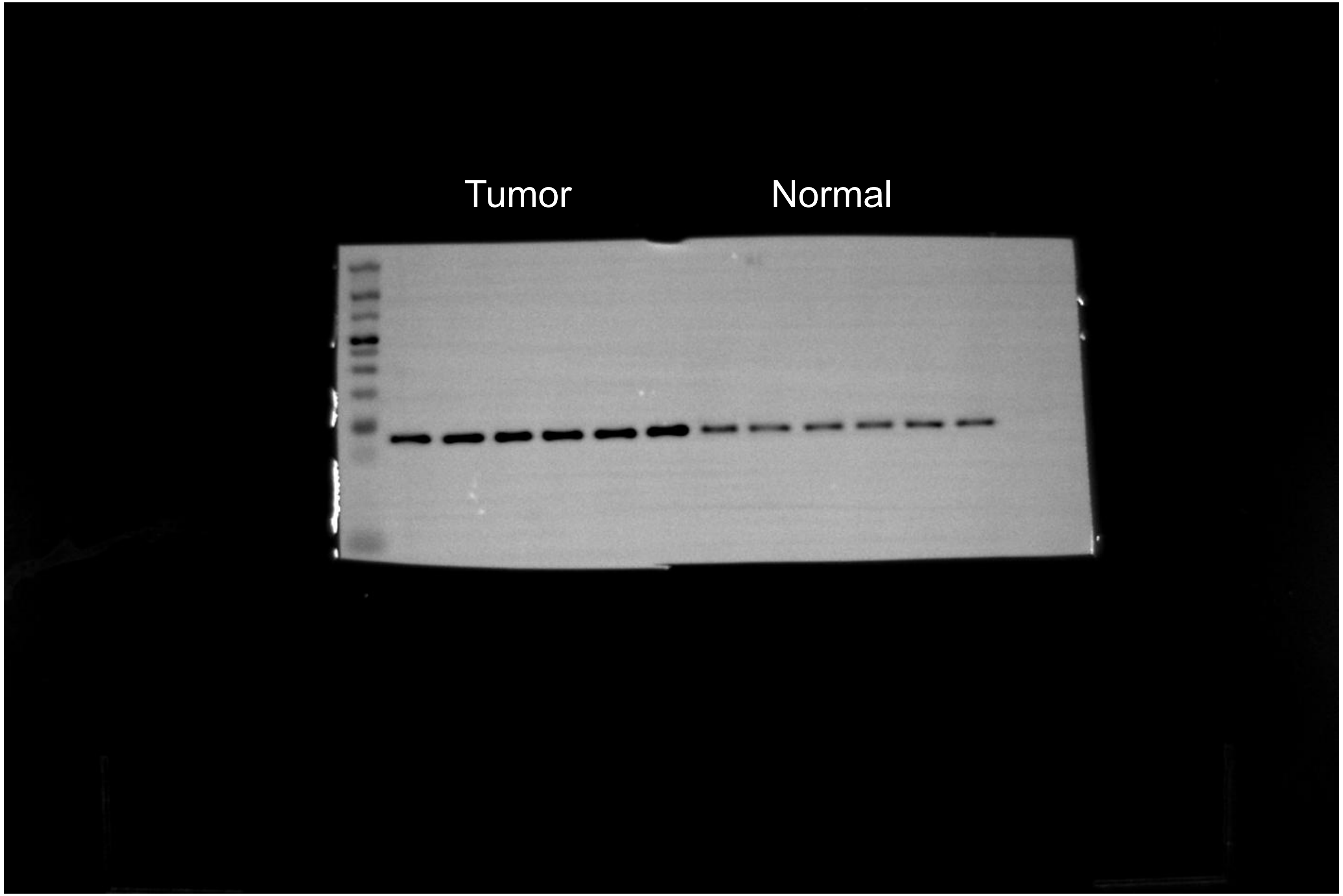

FAT1

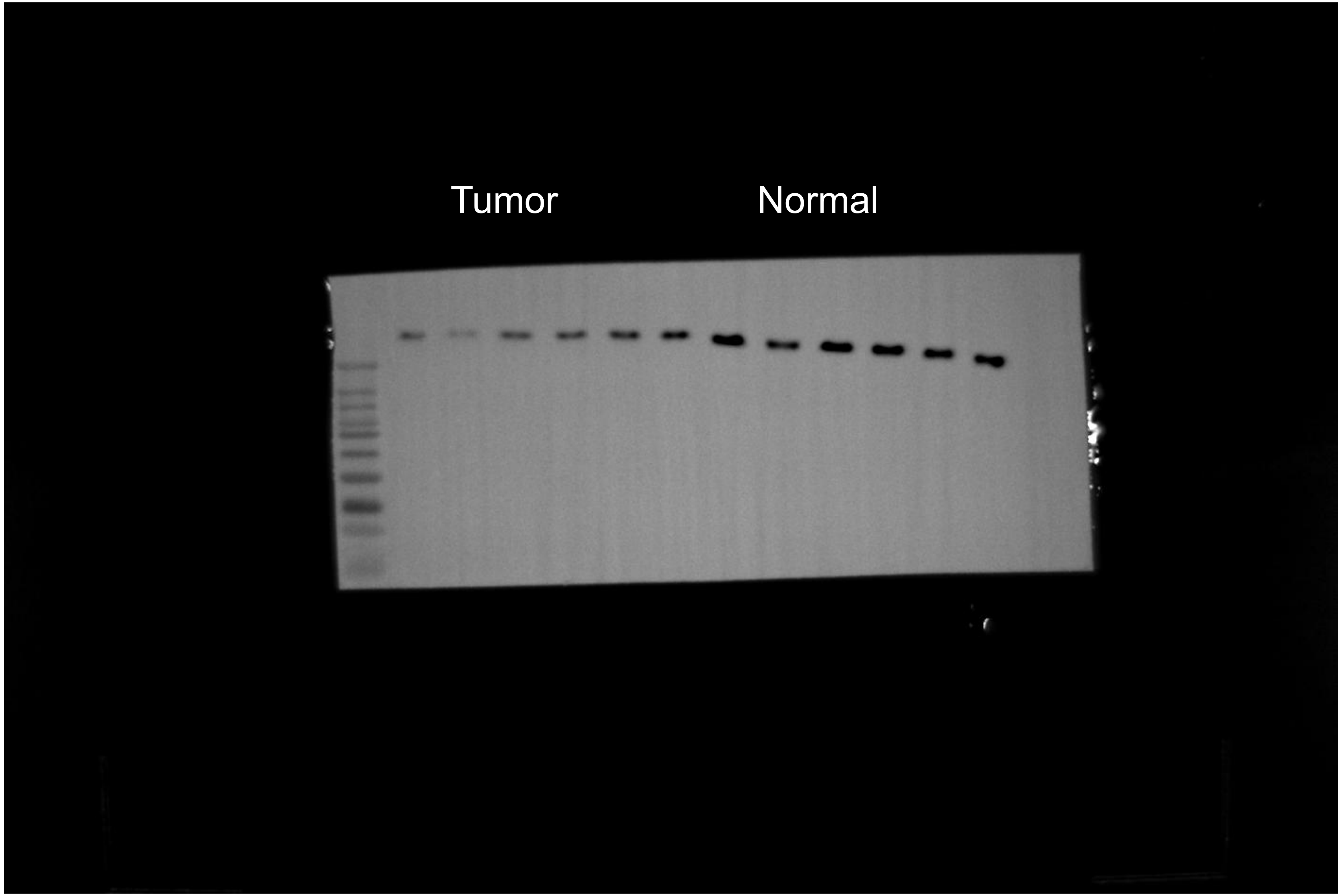

GAPDH

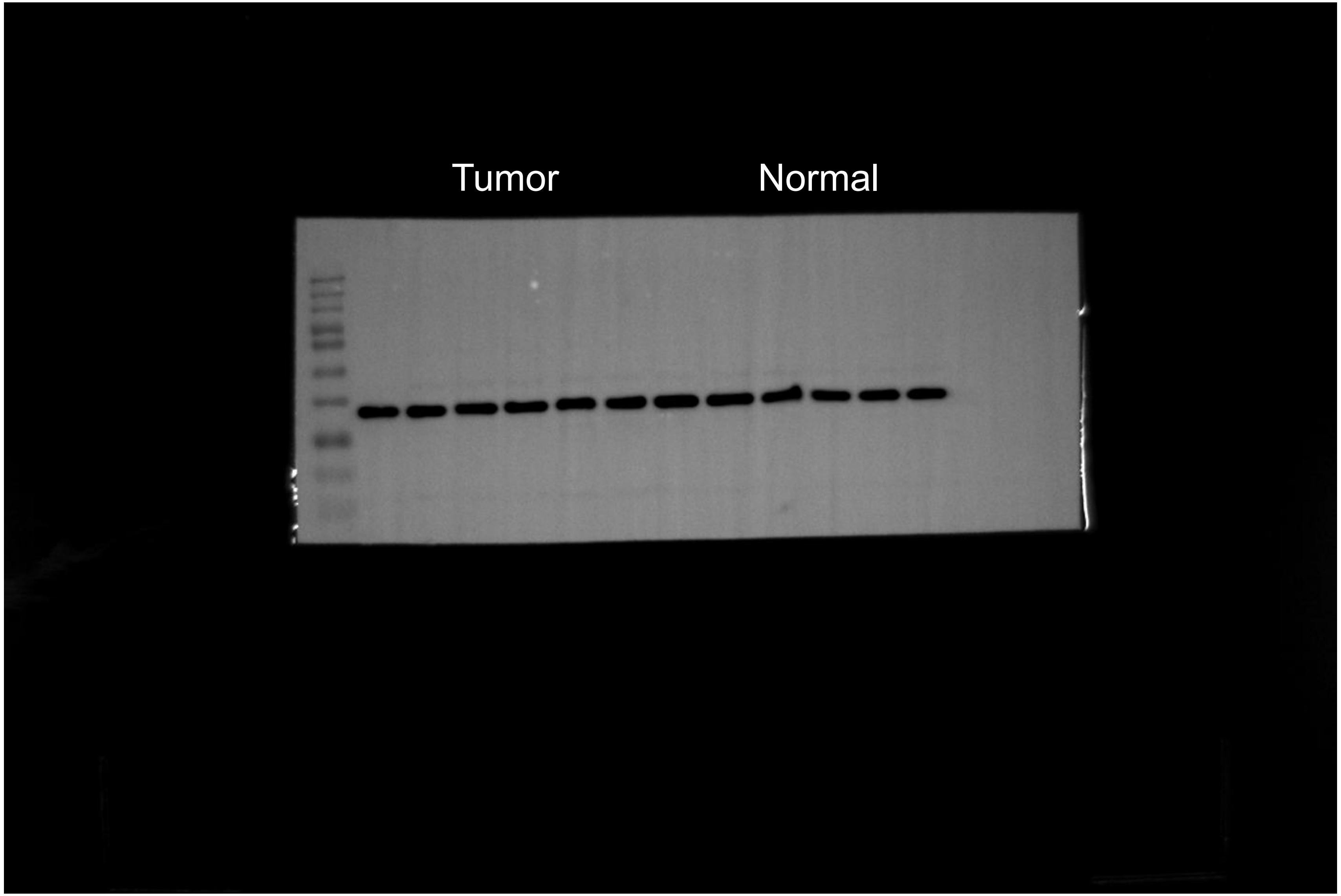

MTIE

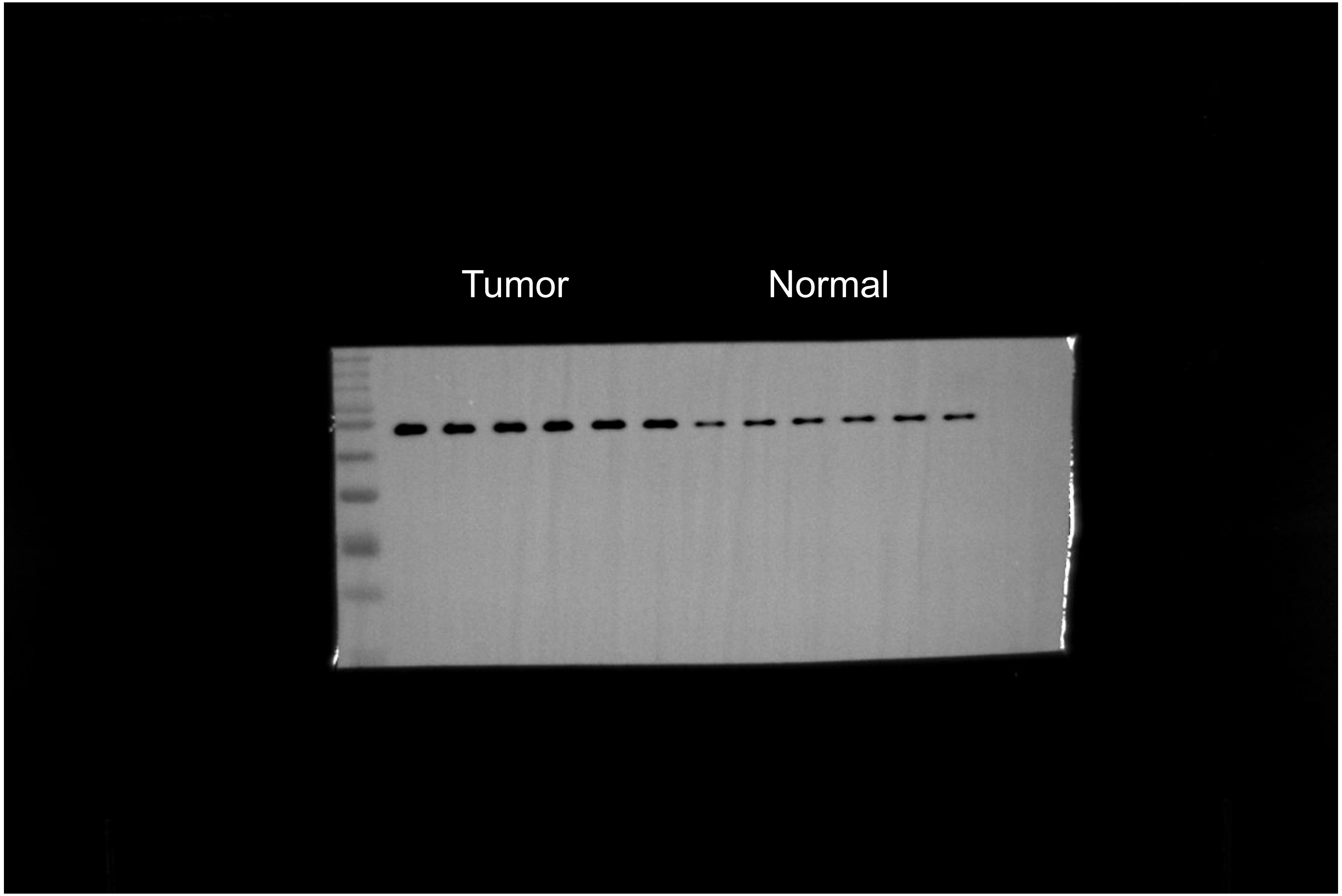

MTIX

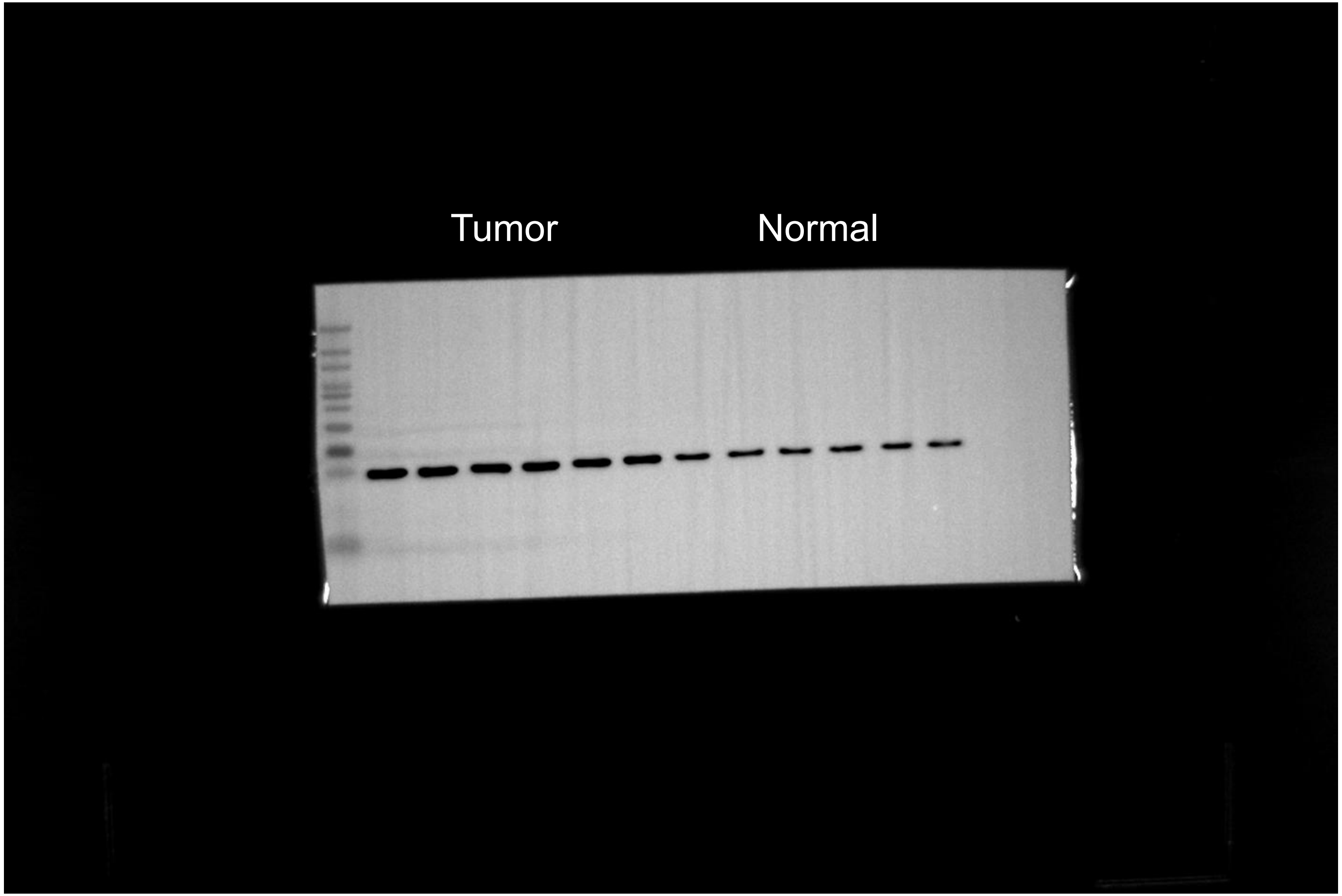

OAS2

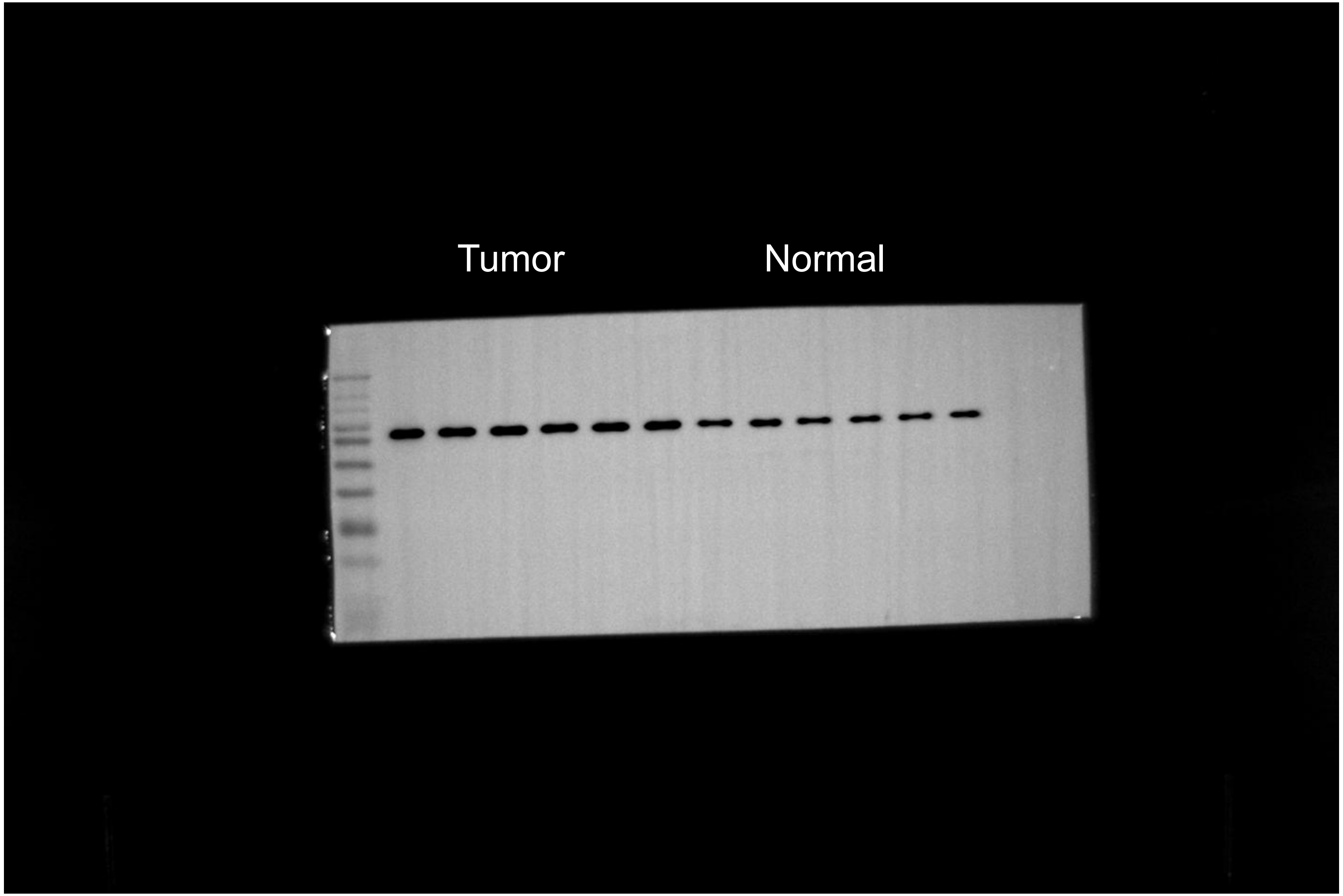

TNFRSF1

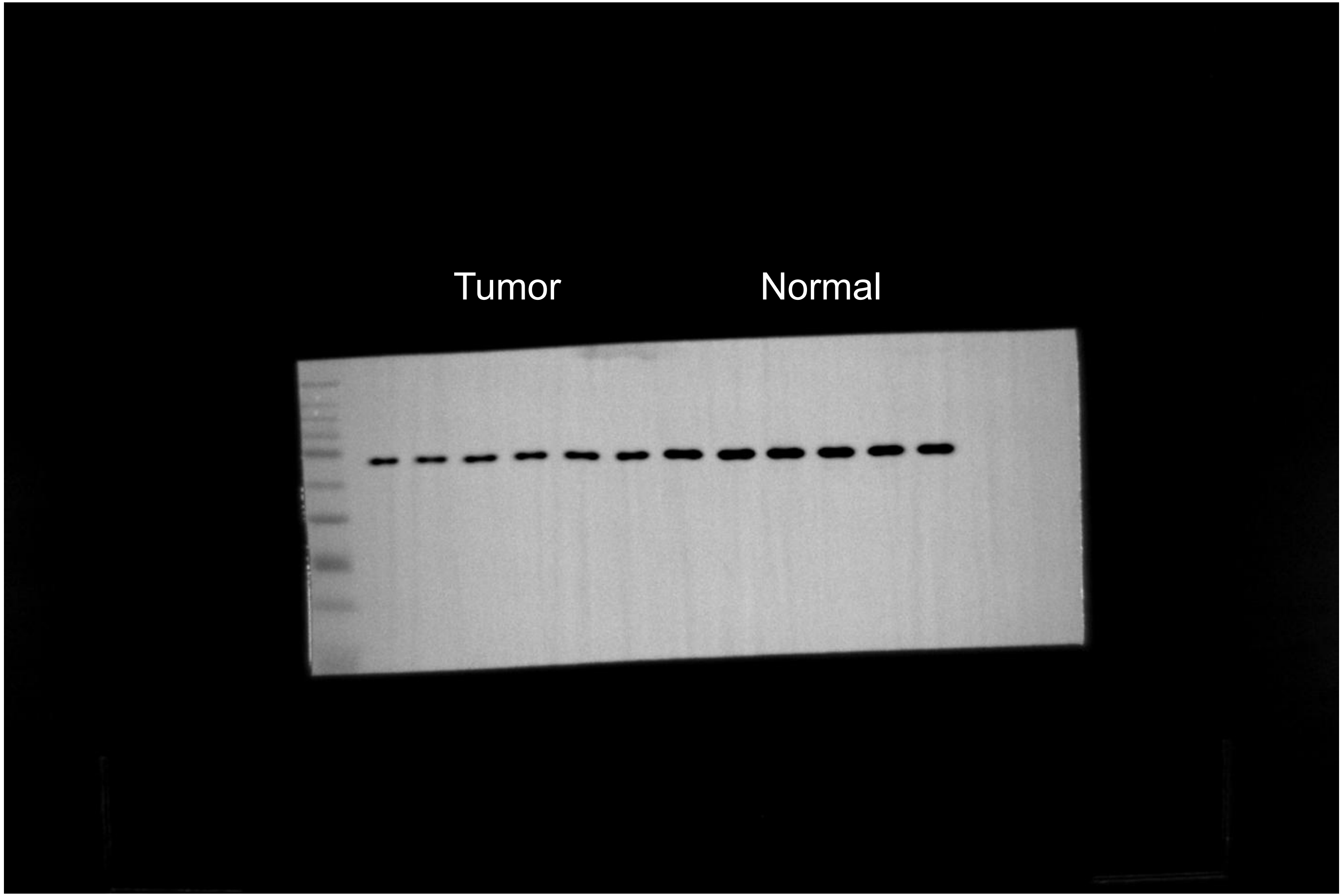

Supplement: Supplementary file 7 [file DataSheet7.pdf]
