# Supplementary material for: Effect of immune infiltration intensity on the efficacy of neoadjuvant immunotherapy for esophageal cancer
Source: Front Immunol. 2025 Jun 12;16:1543283. doi: 10.3389/fimmu.2025.1543283 (PMC12198219; doi:10.3389/fimmu.2025.1543283)
Supplement: Supplementary file 8 [file Table1.docx]

| **Data Type** | **Data Source** | **Reference** |
| --- | --- | --- |
| Single-cell RNA sequencing data | Collected from 22 patients with resectable esophageal squamous cell carcinoma (ESCC) before and after neoadjuvant therapy. Derived from the single-cell data of a previous study and subjected to conventional cell annotation analysis. | Li C, Song W, Zhang J, Luo Y. Single - cell transcriptomics reveals heterogeneity in esophageal squamous epithelial cells and constructs models for predicting patient prognosis and immunotherapy. Front Immunol 2023, 14:1322147. doi:10.3389/fimmu.2023.1322147 |
| RNAseq data | 182 esophageal carcinoma (ESCA) samples and 13 normal samples from The Cancer Genome Atlas (TCGA) database | [https://portal.gdc.cancer.gov/](https://portal.gdc.cancer.gov/) |

Table S1. Data Source

#Data

#Single-cell RNA sequencing data doi:10.3389/fimmu.2023.1322147

#RNAseq data https://portal.gdc.cancer.gov/

#Part of the code

#1

library(Seurat)

escc_data <- Read10X(data.dir = "path/to/your/data")

escc_seurat <- CreateSeuratObject(counts = escc_data)

escc_seurat <- NormalizeData(escc_seurat)

escc_seurat <- FindVariableFeatures(escc_seurat)

gene_expression <- FetchData(escc_seurat, vars = c("CXCL10", "CXCL11", "MAGEA1", "OAS2", "CD209", "CD79A", "KLRB1", "TNFRSF17"))

#2

library(DESeq2)

countData <- read.csv("path/to/your/countData.csv")

colData <- read.csv("path/to/your/colData.csv")

dds <- DESeqDataSetFromMatrix(countData = countData, colData = colData, design = ~ condition)

dds <- DESeq(dds)

res <- results(dds, name = "condition_treatment_vs_control")

#3

library(maftools)

maf_data <- read.maf(maf = "path/to/your/maf_file.maf")

tmb <- tmb(maf_data)

genes_of_interest <- c("CXCL10", "CXCL11", "MAGEA1", "OAS2", "CD209", "CD79A", "KLRB1", "TNFRSF17")

gene_mut_data <- subset(maf_data@data, Hugo_Symbol %in% genes_of_interest)

correlation_result <- cor.test(gene_mut_data$TMB, gene_mut_data$Expression, method = "pearson")

#4

library(survminer)

survival_data <- read.csv("path/to/your/survival_data.csv")

median_expression <- median(survival_data$gene_expression)

survival_data$expression_group <- ifelse(survival_data$gene_expression >= median_expression, "high_expression", "low_expression")

surv_obj <- Surv(time = survival_data$survival_time, event = survival_data$survival_status)

km_fit <- survfit(surv_obj ~ expression_group, data = survival_data)

ggsurvplot(km_fit, data = survival_data, risk.table = TRUE)

#5

gene_name <- "CXCL10"

group_info <- c("high_expression", "low_expression")

ic50_result <- send_request_to_GEPIA2(gene_name, group_info)

plot_ic50_result(ic50_result)

#6

qrtpcr_data$relative_expression <- 2^(-(qrtpcr_data$tumor_ct - qrtpcr_data$normal_ct))

qrtpcr_data$normalized_expression <- qrtpcr_data$relative_expression / qrtpcr_data$gapdh_expression
