# Supplementary material for: Effect of immune infiltration intensity on the efficacy of neoadjuvant immunotherapy for esophageal cancer
Source: Front Immunol. 2025 Jun 12;16:1543283. doi: 10.3389/fimmu.2025.1543283 (PMC12198219; doi:10.3389/fimmu.2025.1543283)
Supplement: Supplementary file 9 [file Table2.docx]

Table S2 Differentially Expressed mRNA Genes and Their Roles in Immune Activity

| mRNA | Role in Immune Activity |
| --- | --- |
| CXCL10 | Binding of this protein to CXCR3 results in pleiotropic effects, including stimulation of monocytes, natural killer and T-cell migration, and modulation of adhesion molecule expression. |
| CXCL11 | Chemokines are a group of small (approximately 8 to 14 kD), mostly basic, structurally related molecules that regulate cell trafficking of various types of leukocytes through interactions with a subset of 7-transmembrane, G protein-coupled receptors |
| MAGEA1 | May play a role in embryonal development and tumor transformation or aspects of tumor progression. Antigen recognized on a melanoma by autologous cytolytic T-lymphocytes. |
| OAS2 | This gene encodes a member of the 2-5A synthetase family, essential proteins involved in the innate immune response to viral infection. |
| CD209 | This gene encodes a C-type lectin that functions in cell adhesion and pathogen recognition. Diseases associated with CD209 include Dengue Virus and Human Immunodeficiency Virus Type 1. Among its related pathways are Infectious disease and Respiratory Syncytial Virus Infection Pathway. |
| CD27 | The protein encoded by this gene is a member of the TNF-receptor superfamily. This receptor is required for generation and long-term maintenance of T cell immunity. It binds to ligand CD70, and plays a key role in regulating B-cell activation and immunoglobulin synthesis. |
| CD79A | The B lymphocyte antigen receptor is a multimeric complex that includes the antigen-specific component, surface immunoglobulin (Ig). Surface Ig non-covalently associates with two other proteins, Ig-alpha and Ig-beta, which are necessary for expression and function of the B-cell antigen receptor. This gene encodes the Ig-alpha protein of the B-cell antigen component. |
| KLRB1 | Natural killer (NK) cells are lymphocytes that mediate cytotoxicity and secrete cytokines after immune stimulation. |
| TNFRSF17 | The protein encoded by this gene is a member of the TNF-receptor superfamily. This receptor is preferentially expressed in mature B lymphocytes, and may be important for B cell development and autoimmune response. |
